# High‐frequency ultrasound findings of sebaceous carcinoma in the eyelid

**DOI:** 10.1111/srt.13555

**Published:** 2024-01-04

**Authors:** Guiwu Chen, Xiaoling Leng, Wenqin Liu, Jiaxin Meng, Xiaomin Liao

**Affiliations:** ^1^ Department of Ultrasound The Tenth Affiliated Hospital of Southern Medical University, Dongguan people's hospital Dongguan China; ^2^ Department of Pathology The Tenth Affiliated Hospital of Southern Medical University, Dongguan people's hospital Dongguan China

Dear editor,

Sebaceous carcinoma (SC), a rare malignant tumor arising from the sebaceous glands’ adnexal epithelium, accounts for 0.7% of skin malignancy. Typically, SCs present as nonspecific and painless nodules with pink or yellow appearance.^1^ Due to their low incidence, SCs are often missed or misdiagnosed. Here, we report a case of SC in the eyelid that was characterized by high‐frequency ultrasound and confirmed by pathological examination.

A 50‐year‐old female presented to our hospital with a history of a mass in the eyelid for half a year without any symptoms. Upon physical examination, a yellow mass was observed on the outer aspect of the upper right eyelid. This mass had a cauliflower‐like shape, a hard texture, poor mobility, and unclear boundaries. The eyelid margin was thickened and slightly eroded with a small amount of secretion. Laboratory examination revealed cancer antigen 125 (CA125) of 12.7 U/mL, cancer antigen 15‐3 (CA15‐3) of 9.1 U/mL, Alpha‐fetoprotein (AFP) of 4.94 ng/mL, carcinoembryonic antigen (CEA) of 0.88 ng/mL, and cancer antigen 19‐9 (CA19‐9) of 10.3 U/mL.

High‐frequency ultrasound revealed that the solid mass located in the right eyelid was inhomogeneous and hypoechoic with abundant blood flow signals inside the mass (Figure 1). Magnetic resonance imaging examination revealed that the nodular mass had iso‐intensity on both T1‐weighted and T2‐weighted images, and obvious enhancement was observed on the contrast‐enhanced magnetic resonance imaging (Figure 2). Finally, the patient underwent a surgical resection of the mass, and pathological examination confirmed the diagnosis of SC. Hematoxylin and eosin (H&E) staining showed proliferation and infiltration of abnormal cells in the superficial layer of the dermis (Figure 3). Immunohistochemical staining results were positive for EMA, CK7, CK5/6, and Ki‐67 (approximately 90%), but negative for CEA.

**FIGURE 1:**
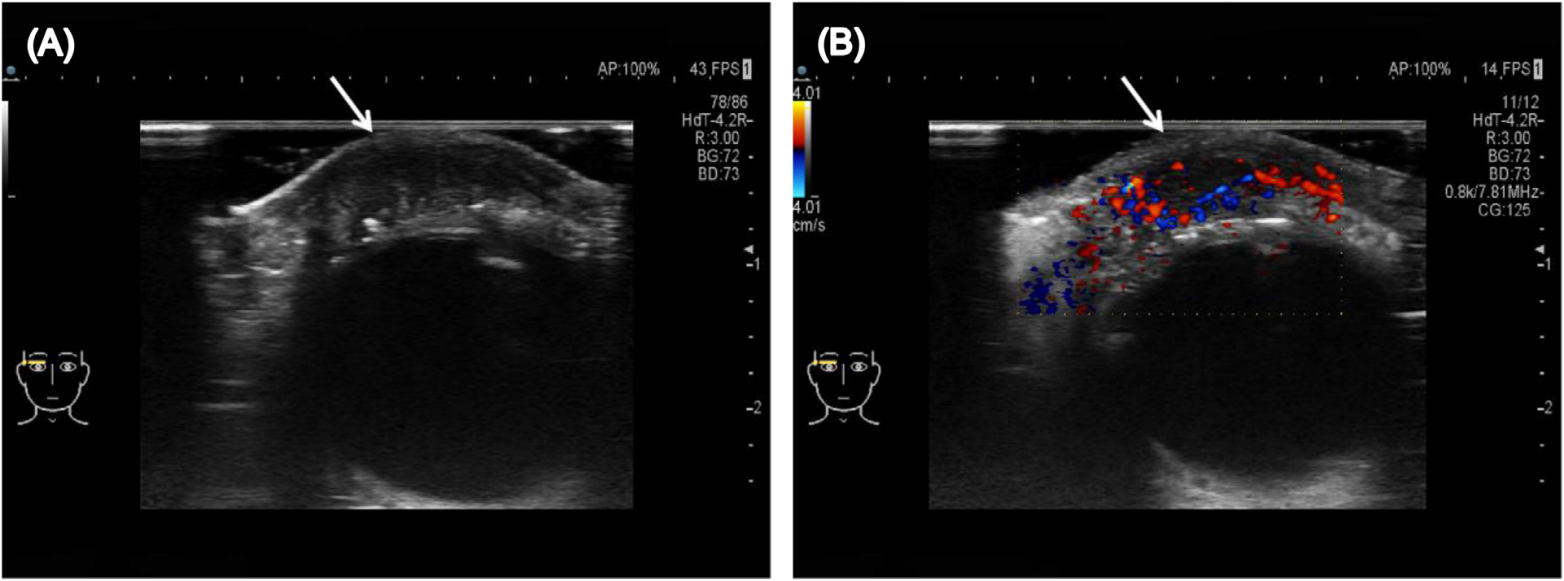
High‐frequency ultrasound of sebaceous carcinoma. (A) Grayscale ultrasound showed the solid mass located in the right eyelid was inhomogeneous, hypoechoic, ill‐defined, and irregular. (B) Color Doppler flow imaging showed abundant blood flow signals inside the mass.

**FIGURE 2:**
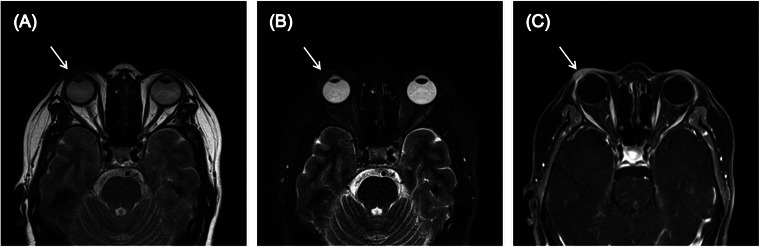
Magnetic resonance imaging of sebaceous carcinoma. (A) The nodular mass had iso‐intensity on T1‐weighted images. (B) The nodular mass had iso‐intensity on T2‐weighted images. (C) The nodular mass had obvious enhancement on the contrast‐enhanced MRI.

**FIGURE 3:**
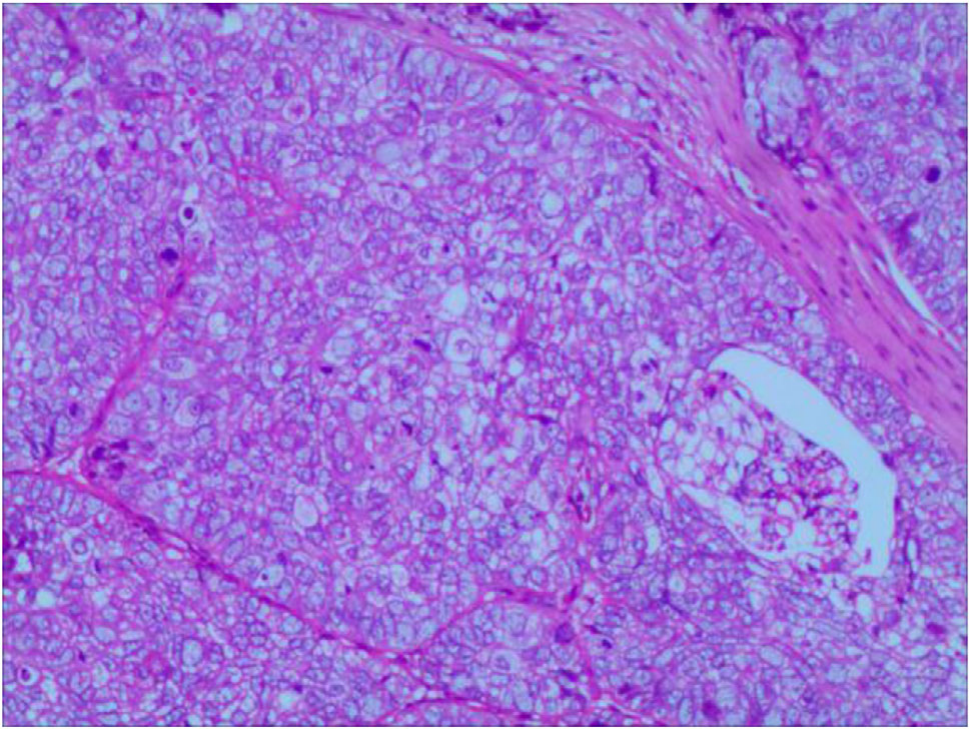
Pathological examination of sebaceous carcinoma. Hematoxylin and eosin staining showed proliferation and infiltration of abnormal cells in the superficial layer of the dermis, with rich cytoplasm, foam‐like appearance, large nucleus, deep staining, and significant atypia.

SCs are characterized by slow growth, and most patients with SC present late with an advanced tumor and distant metastasis, carrying a 30%–40% risk of local tumor recurrence, a 20%–25% risk of distant metastasis, and a 10%–30% risk of tumor‐related mortality.^1^, ^2^ High‐frequency ultrasound has high resolution and the ability to clearly display the structures of skin, which is advantageous for the diagnosis and follow‐up of skin tumors.^3^ In our case, high‐frequency ultrasound showed SC was inhomogeneous, hypoechoic, ill‐defined, and irregular with abundant blood flow signals. However, the evidence is still insufficient to differentiate SC from other similar skin tumors, and further research should be undertaken.

## CONFLICT OF INTEREST STATEMENT

Our research group declares no conflict of interest.

## Data Availability

The data used to support the findings of this study are available from the corresponding author upon request.
